# Pain relieving effect of dexmedetomidine in patients undergoing total knee or hip arthroplasty: A meta-analysis

**DOI:** 10.1097/MD.0000000000018538

**Published:** 2020-01-03

**Authors:** Qi Yang, Yi Ren, Bin Feng, Xisheng Weng

**Affiliations:** a Department of Orthopedics, Peking Union Medical College Hospital, Chinese Academy of Medical Sciences and Peking Union Medical College, No. 1 Shuaifuyuan Wangfujing, Dongcheng District, Beijing; b Department of Orthopedics, First Hospital of Harbin, Harbin, China.

**Keywords:** delirium, dexmedetomidine, meta-analysis, pain management, total hip arthroplasty, total knee arthroplasty

## Abstract

**Background:**

To evaluate the safety and efficacy of dexmedetomidine in patients undergoing total knee and hip arthroplasty for postoperative pain control.

**Methods:**

An updated systematic review and meta-analysis of randomized controlled trials (RCTs) identified in systematic searches of MEDLINE, EMBASE, Google Scholar, the Cochrane Database and the Chinese SinoMed Database.

**Results:**

Fourteen RCTs with a total of 1220 patients were included. Overall, dexmedetomidine therapy was associated with significantly decreased pain scores 24 hours after surgery (WMD, −0.36; 95% CI, −0.49 to −0.22; I^2^ = 90.0%, *P* < .001) compared with scores in the control group after total hip arthroplasty (THA) and total knee arthroplasty (TKA). Furthermore, the rate of postoperative delirium was also markedly decreased with dexmedetomidine therapy (RR, 0.38; 95% CI, 0.24 to 0.59; I^2^ = 0.0%, *P* < .001). Moreover, compared with the control group, dexmedetomidine treatment was associated with a decreased risk of postoperative nausea and vomiting in patients undergoing TKA (RR, 0.34; 95% CI, 0.15 to 0.79; I^2^ = 0.0%, *P* = .012), and there was a similar risk of hypotension (RR, 1.03; 95% CI, 0.72 to 1.49; I^2^ = 24.4%, *P* = .87) regardless of whether patients underwent TKA or THA. However, the rate of bradycardia was significantly increased with dexmedetomidine treatment in those undergoing TKA (RR, 6.11; 95% CI, 2.35 to 15.91; I^2^ = 0.0%, *P* < .001).

**Conclusions:**

Dexmedetomidine therapy seems to be an effective treatment for pain control and postoperative delirium in patients undergoing TKA/THA. However, the incidence of bradycardia is markedly increased in patients undergoing TKA. Hence, much larger prospective clinical studies are warranted to confirm these findings.

## 1 Introduction

Patients who undergo total knee or hip arthroplasty are associated with significant postoperative pain,^[1,2]^ which may be underestimated. Previous studies have found that postoperative pain can affect early mobilization and psychological state and can delay patient discharge and early rehabilitation in patients with knee arthroplasty.^[3,4]^ Therefore, adequate pain management is very important to reduce morbidity and promote recovery.^[5]^

Many agents have been used to provide effective postoperative pain control, including local anesthetics such as lidocaine and bupivacaine, opioids such as morphine, and α2-adrenergic receptor agonists such as clonidine.^[5]^ However, none of these anesthetics are free from limitations such as the need for special equipment and monitoring or the risk of complications that may delay discharge or cause readmission.

Dexmedetomidine is a highly selective, specific, and potent α2-adrenergic receptor agonist.^[6]^ Many studies have demonstrated that dexmedetomidine can significantly decrease pain scores and postoperative diclofenac sodium consumption and can improve the duration of the analgesic effect.^[7,8]^ Furthermore, the incidence of postoperative delirium^[9]^ and postoperative nausea and vomiting (PONV)^[10]^ decreases with the use of dexmedetomidine therapy. However, several studies have shown that dexmedetomidine administration is associated with an increased risk of hypotension^[11]^ and bradycardia requiring atropine.^[12]^ Therefore, the safety and efficacy of dexmedetomidine therapy in patients undergoing total knee arthroplasty (TKA) and total hip arthroplasty (THA) is still debatable. Moreover, previous studies are limited by a small size and low quality. Therefore, as the amount of evidence has been recently increasing, we performed a meta-analysis to evaluate the safety and efficacy of dexmedetomidine therapy in patients with TKA/THA.

## 2 Materials and methods

### 2.1 Data sources and search strategies

We searched MEDLINE (source, PubMed from 2005 to April 2018), EMBASE (2005 to April 2018), the Cochrane Controlled Clinical Trials Register Database (to April 2018), and the ClinicalTrials.gov website (to April 2018) using the terms “total knee arthroplasty”, “total hip arthroplasty”, “dexmedetomidine”, “pain intensity”, “pain score”, “delirium”, and “randomized trial” in each database. Manual reference checking of the bibliographies of all relevant articles was performed. The reference list of relevant studies was additionally screened. No language restrictions were applied. There is no need for an approval from ethics committee because our study belongs to a retrospective type focusing on literature review.

### 2.2 Study selection

We first conducted an initial screening of titles and abstracts; the second evaluation was based on a full-text review. Trials were considered eligible if they met the following criteria:

1) the study was a prospective randomized controlled trial (RCT);
2) the intervention consisted of dexmedetomidine treatment; and
3) the primary outcome of interest was the change in pain intensity.

The exclusion criteria were

1) non-RCTs and
2) duplicate data.

### 2.3 Data extraction

Two reviewers (QY and YR) extracted data concerning patient characteristics, dexmedetomidine use, study quality, and clinical outcomes using a standard data-collection form. Disagreements were resolved via discussion by 2 reviewers (QY and YR) and the corresponding author (XSW).

The primary outcome was the change in pain intensity at rest and upon movement 24 hours postoperatively. The secondary outcomes were the rate of postoperative delirium, dose of opioid consumption, duration of the analgesic effect, and time to first analgesic request. Adverse events included PONV, hypotension, bradycardia, and somnolence.

### 2.4 Quality assessment

The Preferred Reporting Items for Systemic Reviews and Meta-Analyses (PRISMA) statement^[13]^ was followed. Two reviewers (QY and YR) assessed the quality of the selected trials based on the Jadad Scale^[14]^ and the Cochrane Collaboration method.^[15]^ The components used for quality assessment were randomization (0–2 points), blinding (0–2 points), dropouts and withdrawals (0–1 points).

### 2.5 Data synthesis and analysis

The results were analyzed quantitatively with STATA 14.0 software (Stata Corp, CA) using the random-effects model^[16]^ in consultation with one statistician. We calculated the pooled relative risk (RR) for dichotomous outcomes and the weighted mean difference (WMD) for continuous data with 95% confidence intervals (CIs).

Heterogeneity was examined with the I^2^ statistic and the chi-squared test. A value of I^2^ > 50% was considered a substantial level of heterogeneity.^[17]^ Once heterogeneity was noted, publication bias was assessed quantitatively using Egger regression test (*P* ≤ .10)^[18]^ and qualitatively with the visual inspection of funnel plots of the logarithm of RRs versus their standard errors.^[19]^ Sensitivity analyses were conducted to determine the influence of individual trials on the overall pooled results. All analyses were performed according to the intention-to-treat principle. Statistical significance was set at *P* < .05.

## 3 Results

### 3.1 Search results

**Figure 1:**
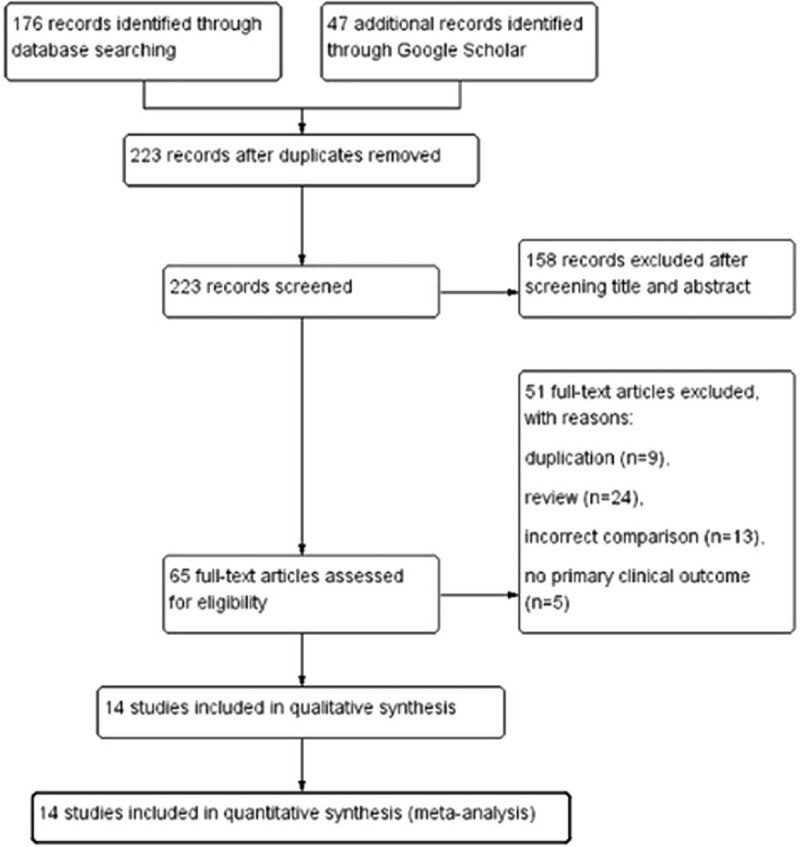
Flow chart.

We initially identified 223 potentially relevant articles. Sixty-five studies were considered to be of interest and were retrieved for full-text review. Fifty-one articles were excluded due to duplication (n = 9), reviews (n = 24), incorrect comparisons (n = 13) and a lack of primary clinical outcomes (n = 5). Therefore, 14 randomized trials including 1 study identified in the references were finally included in the analysis. Figure 1 is a flow chart showing the study selection process.

### 3.2 Study characteristics

**Figure 2:**
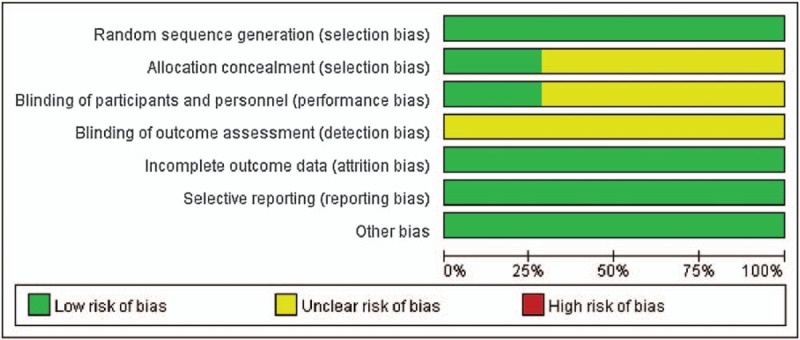
Risk of bias.

**Figure 3:**
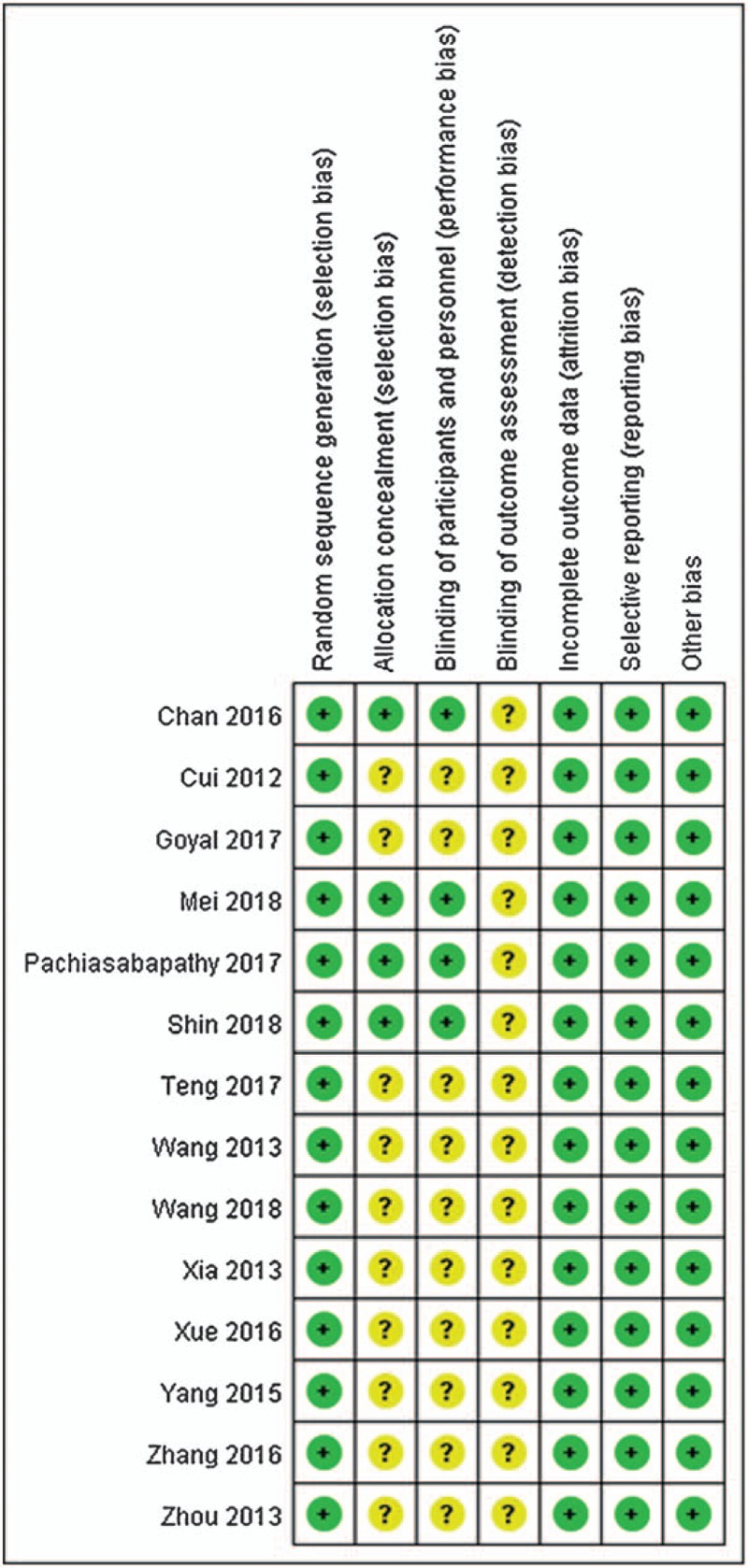
Risk of bias evaluation.

Fourteen^[20–33]^ published RCTs with a total of 1220 patients were included. The total number of patients in each study ranged from 40 to 296. The participants’ ages ranged from 72.3 ± 12.5 years. Most patients were male, with a percentage of 64.3%. The mean duration of surgery was 78.6 minutes. All patients in the treatment group were treated with dexmedetomidine alone (0.5–2 ug/kg) or in combination with ropivacaine, bupivacaine, or propofol. Patients in the control group were treated with saline, ropivacaine, bupivacaine or propofol. Two studies had thee comparisons because of different dexmedetomidine concentrations. The Jadad scores of all included studies varied from 3 to 5; all 14 studies were considered to be of high quality according to the quality assessment (Table 1, Figs. 2 and 3).

**Table 1:**
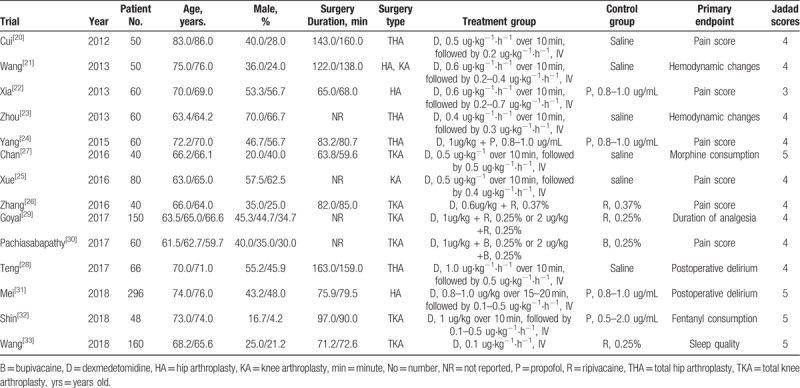
Characteristics of patients with dexmedetomidine therapy in knee or hip arthroplasty.

### 3.3 Efficacy of postoperative pain management

**Figure 4:**
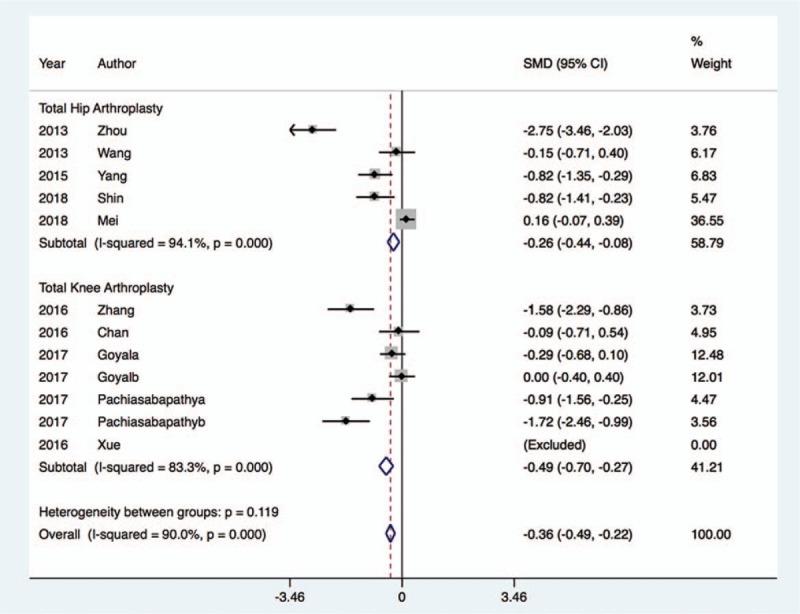
Change of pain intensity evaluated by VAS after dexmedetomidine treatment.

**Figure 5:**
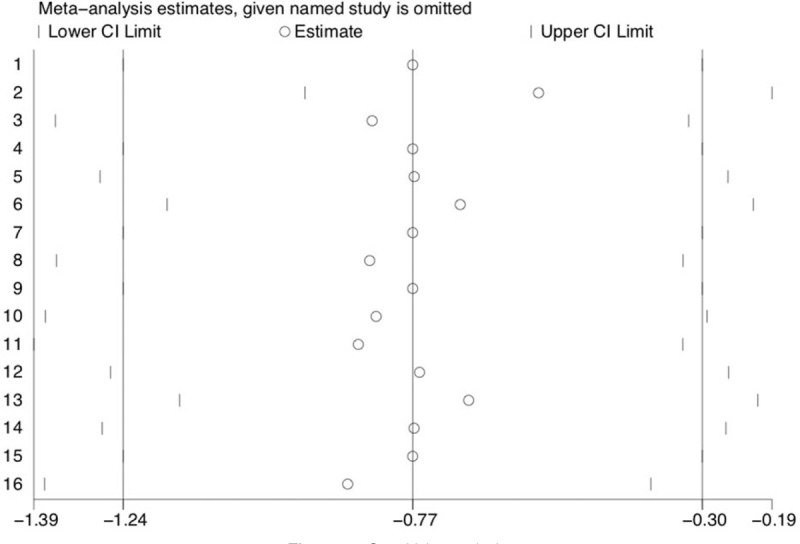
Sensitivity analysis.

Overall, 10 of 14 trials^[21,23–27,29–32]^ provided data about pain intensity 24 hours postoperatively, with 642 patients in the dexmedetomidine group and 647 patients in the control group. Compared with the control group, dexmedetomidine therapy was associated with significantly decreased pain scores 24 hours after surgery (WMD, −0.36; 95% CI, −0.49 to −0.22; I^2^ = 90.0%, *P* < .001), regardless of whether the patients underwent THA (WMD, −0.26; 95% CI, −0.44 to −0.08; I^2^ = 94.1%, *P* < .01) or TKA (WMD, −0.49; 95% CI, −0.70 to −0.27; I^2^ = 83.3%, *P* < .001) (Fig. 4). However, there was a high level of heterogeneity (I^2^ = 90.0%). The funnel plot showed marked asymmetry according to Begg's test (*P* = .03) and Egger test (*P* = .02). However, sensitivity analysis was performed by removing each of the trials one at a time, which did not detect any influence on the change in pain intensity (Fig. 5).

### 3.4 Safety and side effects

**Figure 6:**
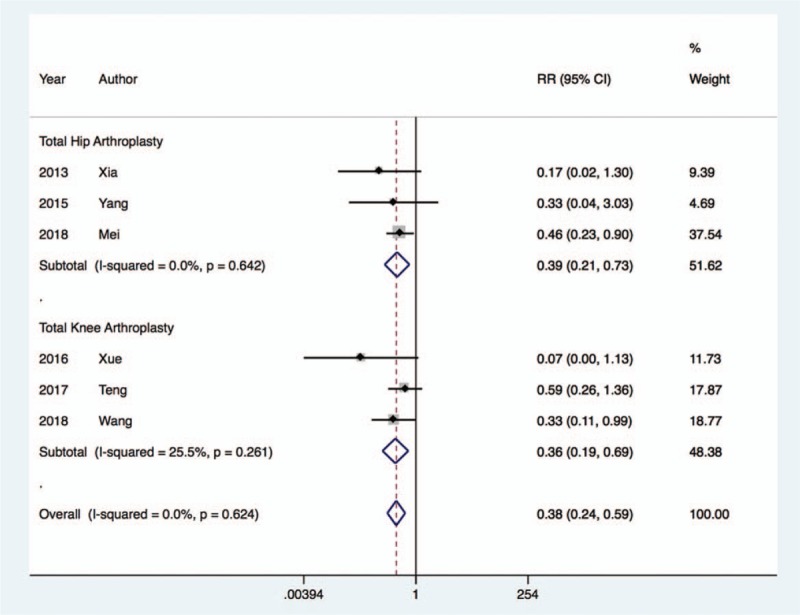
Risk of delirium after dexmedetomidine treatment.

**Figure 7:**
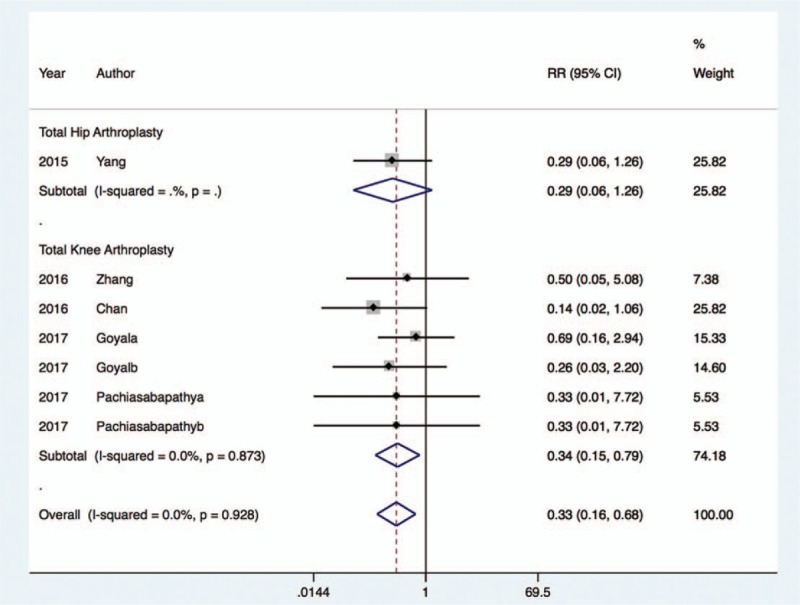
Risk of PONV after dexmedetomidine treatment.

The rate of postoperative delirium^[22,24,25,28,31,33]^ was markedly decreased with dexmedetomidine therapy (RR, 0.38; 95% CI, 0.24 to 0.59; I^2^ = 0.0%, *P* < .001), regardless of whether the patients underwent THA (RR, 0.39; 95% CI, 0.21 to 0.73; I^2^ = 0.0%, *P* < .01) or TKA (RR, 0.36; 95% CI, 0.19 to 0.69; I^2^ = 25.5%, *P* < .01) (Fig. 6). Furthermore, dexmedetomidine treatment was associated with a decreased risk of postoperative nausea and vomiting^[24,26,27,29,30]^ in patients undergoing TKA (RR, 0.34; 95% CI, 0.15 to 0.79; I^2^ = 0.0%, *P* < .01), and this result was comparable in patients undergoing THA (RR, 0.29; 95% CI, 0.06 to 1.26; *P* < .01) (Fig. 7).

**Figure 8:**
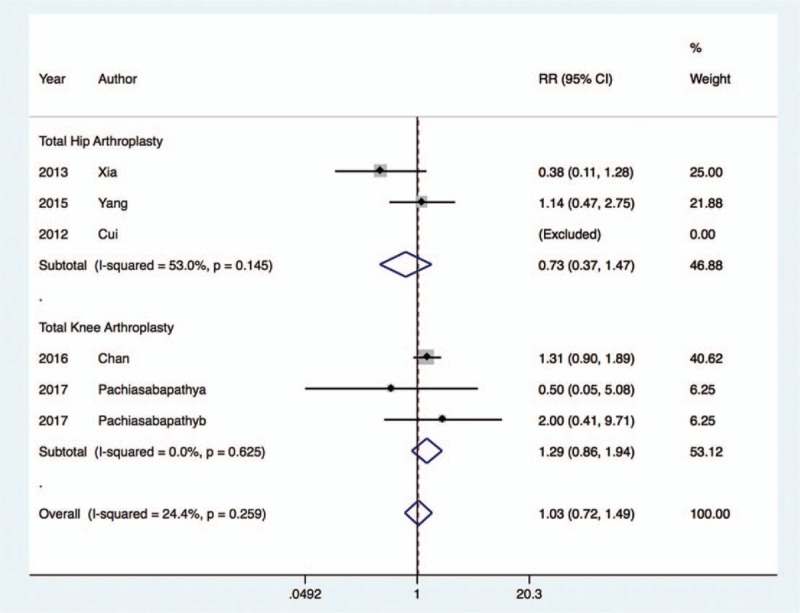
Risk of hypotension after dexmedetomidine treatment.

**Figure 9:**
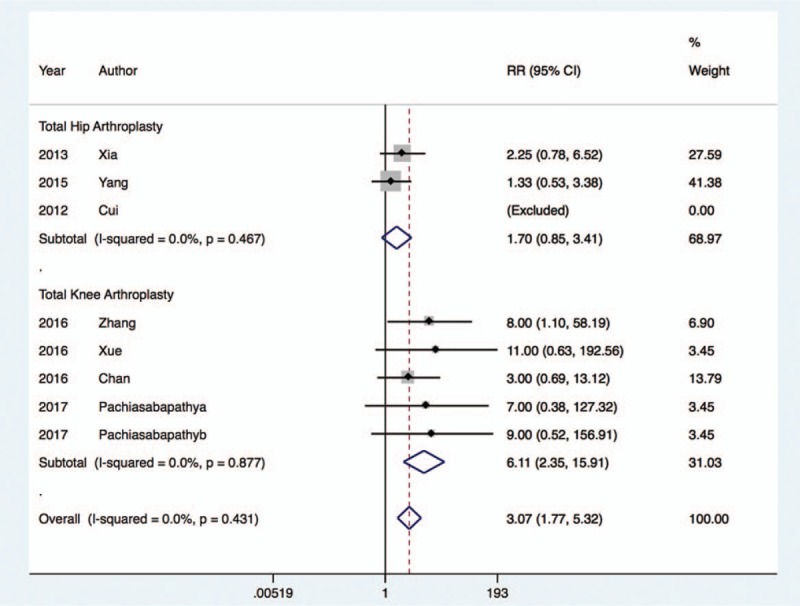
Risk of bradycardia after dexmedetomidine treatment.

The incidence of hypotension^[20,22,24,27,30]^ was similar between the dexmedetomidine group and control group (RR, 1.03; 95% CI, 0.72 to 1.49; I^2^ = 24.4%, *P* = .87), regardless of whether the patients underwent THA (RR, 0.73; 95% CI, 0.37 to 1.47; I^2^ = 53.0%, *P* = .38) or TKA (RR, 1.29; 95% CI, 0.86 to 1.94; I^2^ = 0.0%, *P* = .21) (Fig. 8). Nevertheless, the rate of bradycardia^[20,22,24–27,30]^ was significantly increased with dexmedetomidine treatment in those undergoing TKA (RR, 6.11; 95% CI, 2.35 to 15.91; I^2^ = 0.0%, *P* < .001), and this result was comparable in patients undergoing THA (RR, 1.70; 95% CI, 0.85 to 3.41; I^2^ = 0.0%, *P* = .14) (Fig. 9).

## 4 Discussion

Our meta-analysis of 14 highly qualified randomized controlled clinical trials showed that dexmedetomidine therapy could significantly decrease pain scores compared with placebo in patients undergoing TKA/THA. Furthermore, the risk of postoperative delirium was markedly decreased in the dexmedetomidine group. Moreover, the incidence of PONV was significantly lower in patients undergoing TKA with dexmedetomidine treatment, and the rate of hypotension was similar between the groups. However, the incidence of bradycardia was markedly increased in those undergoing TKA and administered dexmedetomidine.

Postoperative pain is one of the most commonly reported discomforts after TKA/THA surgery and is described as severe by more than half of patients.^[1]^ Although many pain-relieving remedies have been employed as regular measures, it is still a challenge to optimize postoperative pain control. Opioids typically play a large role in the treatment of postoperative pain after TKA, which may result in a high risk of respiratory depression in patients.^[35,36]^ Compared with the opioid-only pain management strategy, a combination of dexmedetomidine with opioids is conducive to decreasing postoperative pain and simultaneously reducing opioid requirements and opioid-related adverse events.^[37]^ Furthermore, dexmedetomidine therapy alone has also yielded promising results.^[38,39]^ In addition, our study confirmed that dexmedetomidine could decrease pain scores in the first 24 hours, which suggests that the analgesic benefits of dexmedetomidine extend beyond its biological half-life of 2 hours. However, the mechanism underlying this long-term analgesic effect remains unclear. A previous study suggested the existence of different action pathways between the sedation and analgesic effects of dexmedetomidine as a possible mechanism.^[40]^ While the effect of dexmedetomidine is mediated by the ascending noradrenergic pathway in the locus coeruleus, the analgesic effect occurs via an α2- adrenergic receptor-dependent descending pathway in the spinal cord.^[40]^ However, the comparison of dexmedetomidine vs opioids for postoperative pain management remains unclear due to limited data.

Of the postoperative complications, delirium is common among elderly patients and is associated with poor clinical outcomes.^[41]^ Many risk factors are related to postoperative delirium, such as exposure to general anesthetics, pain and the postoperative inflammatory response.^[42]^ Preclinical and clinical studies have found that dexmedetomidine attenuates neurotoxicity induced by general anesthetics, improves postoperative analgesia and inhibits the inflammatory response after surgery.^[34,43]^ Several studies have found that the intraoperative use of dexmedetomidine can prevent postoperative delirium, but the data are inconsistent. In pediatric patients undergoing tonsillectomy and cardiac surgery, intraoperative infusion of dexmedetomidine lowers the incidence of emergent delirium.^[44,45]^ For adult patients undergoing cardiac surgery and microvascular free flap surgery, intraoperative dexmedetomidine slightly decreased the incidence of delirium compared with normal saline, although the differences were not statistically significant between the 2 groups, possibly due to the underpowered sample size.^[46]^ Su et al found that prophylactic low-dose dexmedetomidine significantly decreased the occurrence of delirium during the first 7 days after noncardiac surgery.^[47]^ For elderly patients undergoing joint replacement, several studies confirmed that dexmedetomidine treatment during surgery significantly reduced the incidence of postoperative delirium.^[31,48,49]^ Inconsistent with previous studies, our pooled data indicated that dexmedetomidine could markedly lower the risk of postoperative delirium in patients undergoing TKA/THA. However, in a recent study, the use of dexmedetomidine during general anesthesia did not reduce delirium after major noncardiac surgery, including arthroplasty, in elderly patients.^[50]^ Therefore, more studies are warranted to confirm the effect on postoperative delirium.

The present study has a few limitations. First, our study is based on study-level data and as such shares the flaws of original studies. Notwithstanding the value of patient-level meta-analyses, we believe that a meta-analysis of aggregate data can answer this question. Second, our study enrolled patients with different dexmedetomidine concentrations, which are associated with different safeties and efficacies that can impact the therapeutic potential. Moreover, although the inclusion criteria were broad across all 14 studies, slight differences remained in terms of the characteristics of patients and the doses of dexmedetomidine used. We cannot exclude geographical variations. Third, the sample size was inadequate for excluding small differences in outcome between the 2 groups. Therefore, the power of the studies selected was not high. In this sense, our meta-analysis is just a possible indication, and future studies will require larger numbers of patients and careful matching of key clinical and technical variables to definitively quantify the potential effects of dexmedetomidine therapy in patients undergoing TKA/THA.

Given the clinical data, dexmedetomidine therapy appears to provide clinical benefits with respect to postoperative pain control and delirium prevention for patients with TKA/THA. More studies are needed to evaluate the safety of dexmedetomidine treatment for TKA/THA.

## 5 Conclusion

In summary, dexmedetomidine is effective for postoperative pain control, and there is a decreased risk of postoperative delirium in patients undergoing TKA/THA. However, an increased risk of bradycardia is among the side effects of dexmedetomidine.

## Author contributions

**Data curation:** Yi Ren, Xisheng Weng.

**Formal analysis:** Qi Yang, Yi Ren.

**Investigation:** Qi Yang.

**Methodology:** Qi Yang, Bin Feng.

**Resources:** Xisheng Weng, Bin Feng.

**Software:** Qi Yang.

**Supervision:** Xisheng Weng, Bin Feng.

**Validation:** Xisheng Weng, Bin Feng.

**Visualization:** Xisheng Weng.

**Writing – original draft:** Qi Yang, Yi Ren.

**Writing – review & editing:** Yi Ren, Bin Feng.
